# Posttraumatic Stress Disorder and Incident Type 2 Diabetes: Is Obesity to Blame?

**DOI:** 10.1177/2470547019863415

**Published:** 2019-08-30

**Authors:** Jeffrey F. Scherrer, Patrick J. Lustman

**Affiliations:** 1 Department of Family and Community Medicine, Saint Louis University School of Medicine, St. Louis, MO, USA; 2 Harry S. Truman Veterans Administration Medical Center, Columbia, MO, USA; 3 Department of Psychiatry, Washington University School of Medicine, St. Louis, MO, USA; 4 Bell Street Clinic Opioid Addiction Treatment Program, VA St. Louis Health Care System, St. Louis, MO, USA

Commentary on: Scherrer JF, Salas J, Lustman P, et al. Role of obesity in the association between PTSD and incident diabetes. *JAMA Psychiatry*. 2018;75:1189–1198. Scherrer JF, Salas J, Norman SB, et al. Clinically meaningful PTSD improvement and risk for type 2 diabetes. *JAMA Psychiatry*. In Press.

“All wars are fought twice, the first time on the battlefield, the second time in memory”

—Viet Thanh Nguyen

“PTSD is a whole-body tragedy, an integral human event of enormous proportions with massive repercussions.”

—Susan Pease Banitt

## Posttraumatic Stress Disorder and Incident Diabetes

Numerous epidemiological studies have reported a positive association between posttraumatic stress disorder (PTSD) and higher risk for developing type 2 diabetes (T2D). Although several have shown that this association remains after controlling for measured confounding variables,^1,2^ few have discussed the large decrease in the magnitude of association after controlling for obesity, hyperlipidemia, hypertension, psychiatric conditions, and demographics.^3,4^ In a large cohort of Veterans Health Affairs (VHA) patients, results of age-adjusted Cox proportional hazard models indicated that patients with PTSD compared to those without were significantly more likely to develop T2D (hazard ratio = 1.33; 95% confidence interval: 1.08–1.64).^5^ After adjusting for obesity alone, the association was reduced by 50%. No statistical relationship remained after further adjustment for medical and psychiatric comorbidities. In this model, patients with obesity were 3.5 times more likely to develop T2D than those without, a finding that is consistent with the central role of obesity in the risk of T2D development. In fact, patients with obesity were equally likely to develop T2D (21/1000 Person Years [PY]) independent of PTSD. Likewise, in patients without obesity, the incident rate for T2D was 5.8/1000 PY and 6.4/1000 PY among patients with and without PTSD, respectively.^5^ So why do patients with PTSD and other common psychiatric disorders, for example, depression, have a higher risk for T2D compared to patients without these conditions? Their liability appears to rest largely on their propensity to become physically inactive, overweight, and obese. These factors are well-recognized risks in the population. Increasingly, it is recognized that medications (antidepressants and atypical antipsychotics) impose increases in weight and glucose dysregulation that add to risk of incident diabetes.

The good news is that evidence has emerged demonstrating that lifestyle interventions work, lower significantly the risk of developing diabetes, and in persons with established diabetes, slow progression of diabetes. Interventions to address obesity are central to preventing cardiometabolic disease. Lifestyle interventions, that is, changes in dietary practices and moderate intensity (moderate intensity exercise >150 min/week aimed at >5% weight loss) have proven efficacy. The Diabetes Prevention Trial showed that the intensive lifestyle interventions (metformin vs. placebo) was most effective at reducing weight and a 57% decrease in incident T2D.^6^ Longitudinal follow-up data from three large studies of lifestyle intervention for diabetes prevention indicate sustained reductions in risk for T2D: 45% reduction at 7 years in the Da Qing study,^7^ 43% reduction at 7 years in the Finnish Diabetes Prevention Study,^8^ and 27% reduction at 15 years in the US Diabetes Prevention Program outcomes Study.^9^ As a whole, this research clearly identify weight loss as the most powerful tool and one within the patients control that can decrease the risk and slow the progression of T2D.

The degree of difficulty patients experience in changing lifestyle and achieving and maintaining weight loss appears to be greater in patients with versus without PTSD and other psychiatric disorders. PTSD interferes with weight loss efforts^10^ and obesity may interfere with antidepressant effectiveness.^11^ The former may be due to weight gain associated with antidepressants and antipsychotics disproportionately dispensed to those with versus without PTSD (89.5% vs. 34.5%, antidepressants and 33.0% vs. 5.7%, antipsychotics).^5^ The latter phenomenon may be due to obesity-associated inflammation that interferes with antidepressant medication effects.^11^ Another barrier to weight loss in PTSD and depression is a dysregulated physiological stress response (e.g., hypercortisolemia) which contributes to abdominal obesity.^12^

If PTSD develops largely proximate to the time of military service, then it is likely that in many instances, obesity develops subsequent to PTSD onset. If obesity is a consequence of PTSD, then PTSD psychotherapy that achieves large reductions in PTSD symptoms, or spontaneous symptom decrease, might delay weight gain and/or enable weight loss, and thereby reduce risk for cardiometabolic conditions. Unfortunately, as yet there is no compelling evidence that PTSD improvement is followed by weight loss. We know of only one small cohort study of 30 civilians recruited from a commercial weight loss program in which weight loss and PTSD symptoms reductions occurred in tandem.^13^ Yet in a cohort of thousands of patients from the VHA, our analysis revealed that the course of body mass index over one and three years was unrelated to severity of PTSD and unrelated to decreasing PTSD severity over the same time frame (unpublished analysis).

## Large Reduction in PTSD Symptoms, Obesity, and Incident Diabetes

**Figure 1.**
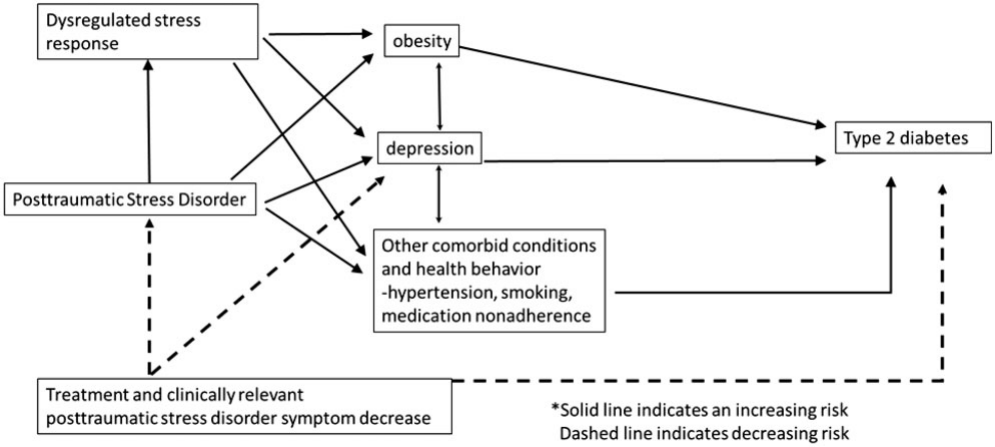
Proposed associations between posttraumatic stress disorder and type 2 diabetes. Solid line indicates an increasing risk. Dashed line indicates a decreasing risk.

We recently observed that Veterans Affairs patients who experienced large clinically significant improvements in PTSD (defined as a ≥20 point decrease on the PTSD Checklist), as compared to those evidencing less than a 20 point improvement, were 49% less likely to develop incident T2D in the following two- to six-year observation period.^14^ Surprisingly, this lower risk was not related to weight change because body mass index and glycemic control remained similar in patients who did and did not experience clinically meaningful PTSD symptom reduction. Only depression improved with PTSD improvement. See Figure 1 for schematic of proposed associations. At first glance, this finding seems to contradict the evidence that obesity largely explains the association between PTSD and incident T2D. However, the prevalence of obesity in patients who did and did not experience clinically meaningful PTSD reduction was 51.3% and 50.1%, respectively, while the prevalence of obesity in the larger population of those with PTSD was 57% and in those without PTSD, 44.8%. Thus, our ability to detect the role of obesity was less when studying patients with PTSD engaged in psychotherapy as compared to evaluating the effect of obesity on risk of T2D in patients with and without PTSD.

## Conclusions

Reducing the cardiometabolic risk associated with PTSD may not depend exclusively on weight loss or improved glycemic control. Our data suggest that PTSD itself may be a modifiable risk factor for diabetes. Aggressive treatment of PTSD that achieves clinically meaningful reduction in PTSD symptom severity may provide a useful stand-alone or adjunctive approach.
